# Prophylactic triple neurectomy during open tension-free inguinal hernia repair: a clinical trial for the prevention of chronic postoperative pain

**DOI:** 10.1007/s13304-025-02497-3

**Published:** 2026-01-22

**Authors:** Yasser A. Orban, Yasmine Hany Hegab, Abd Elwahab M. Hamed, Hasnaa Metwally, Reham Zakaria, Abdalla Mohamed Goda, Yasser Baz

**Affiliations:** 1 Department of General Surgery, Faculty of Medicine, Zagazig University, Zagazig, Egypt; 2 Department of Anesthesia, Intensive Care and Pain Management, Faculty of Medicine, Zagazig University, Zagazig, Egypt; 3 Department of Surgery, Faculty of Medicine, Helwan University (Capital University), Cairo, Egypt

**Keywords:** Inguinal hernia, Neurectomy, Chronic postoperative pain

## Abstract

Postoperative chronic inguinal pain is a common postoperative complication after open inguinal hernia repair e.g. ilioinguinal nerve entrapment is a common cause of chronic pain which may adversely affect the patients’ life. Triple neurectomy (ilioinguinal, iliohypogastric, and genital branch of genitofemoral) seems to be beneficial in preventing such pain, but it may carry the risk for numbness and hypoesthesia. This is a randomized clinical trial, which took place between January 2024 and January 2025, including all patients who were operated on by open inguinal hernioplasty either with triple neurectomy or with nerve preservation. A questionnaire filled by patients either written or by telephone to assess the severity of pain was obtained three months or more after surgery. Fifty one patients were included in nerve preservation group, while 59 patients were included in neurectomy group. Eleven patients in the nerve preservation group suffered from pain of variable severity. Six, four, and one patients had mild, moderate, and sever pain, respectively. In the neurectomy group only three patients had mild pain. There was a significant difference (*p* = 0.008) between the two groups. At time of questionnaire, the number of patients suffered from numbness in the nerve preservation group was 7 patients versus 10 patients in the neurectomy group, which was statistically non-significant, *p* value = 0.641. Prophylactic triple neurectomy provides the advantage of decreasing chronic postoperative pain after open inguinal hernia surgery. While our findings are promising, further studies with larger populations are needed before recommending triple neurectomy as a standard step during inguinal hernioplasty.

***Clinical trial registration***

NCT06327763 registered on Mar 25, 2024.

## Introduction

Open inguinal hernioplasty is one of the most common surgical procedures worldwide [1]. Postoperative chronic inguinal pain (inguinodynia) is a common postoperative complication after open inguinal hernia surgeries, with an incidence that varies between 1 and 23% [1–4]. Inguinodynia is considered if there is persistent pain for more than three months. This chronic inguinal pain can be secondary to nerve entrapment or stretching, inflammation, fibrotic reactions, injury by stitches, or neuroma formation [5, 6].

Nerve entrapment is a common cause of chronic inguinal pain after inguinal hernia surgery. The ilioinguinal nerve is the most entrapped nerve due to its position underneath the external oblique aponeurosis [7]. Conventional surgical education emphasizes the need to protect the nerve during all steps of inguinal hernia repair [8, 9].

Chronic pain after tension-free inguinal hernia repair often affects patients to variable degrees. Mild symptoms have a negligible impact on the patient’s life, but severe symptoms can interfere with normal daily activities and may cause depression, anxiety, and other psychological problems. Thus, postoperative chronic inguinal pain has been consistently considered by surgeons. Several studies have advocated routine ilioinguinal neurectomy to reduce the incidence of postoperative chronic inguinal pain; however, this procedure carries a risk for numbness and hypoesthesia [10–12]. Others recommend ilioinguinal, iliohypogastric, and genitofemoral neurectomy [13]. Thus, routine triple neurectomy is still controversial.

## Methods

Between January 2024 and January 2025, all patients operated on by open inguinal hernioplasty, either with triple neurectomy or with nerve preservation, were included. Group I included 51 patients (nerve preservation group), and group II included 59 patients (triple neurectomy group). This is a randomized prospective clinical trial. Simple randomization was carried out by the sealed envelope method. The actual final sample size was reached after exclusion of patients lost during follow up and those who refused the questionnaire.

This work has been performed in accordance with the Code of Ethics of the World Medical Association (Declaration of Helsinki) for studies involving humans. All procedures were performed in compliance with relevant laws and institutional guidelines and have been approved by the institutional committee.

Informed written consent was obtained from the patients, and they had the right to withdraw from the study at any time without negative impact on their medical care. The results of the study could be used as a scientific publication, but the identity of the participants will be confidential.

Criteria for inclusion: the study included all adult patients operated on for unilateral inguinal hernia by Lichtenstein tension-free mesh hernioplasty with or without triple neurectomy.

Criteria for exclusion: patients with recurrent inguinal hernia, bilateral inguinal hernia repair, or huge inguinoscrotal hernia were excluded. Huge hernias were excluded because they carry a higher risk of developing chronic postoperative edema, a condition known as post-herniorrhaphy lymphedema that may cause heaviness and/or interfere with the daily activities. Female patients were also excluded, as they may have periodic pain (menstrual and ovulatory pain) that may interfere with pain evaluation postoperatively. Diabetic and cardiac patients as well as patients with suspected inguinal hernia recurrence and patients with a BMI more than 35 were excluded.

Patients fasted for 6 h preoperatively and admitted for one-day surgery. Ceftriaxone 1 gm was given IV prior to the incision, and open tension-free Lichtenstein mesh repair was done for all patients under spinal anesthesia. 6 × 11 cm onlay light-weight polypropylene mesh was used and fixed using polypropylene 2/0 sutures.

**Fig. 1:**
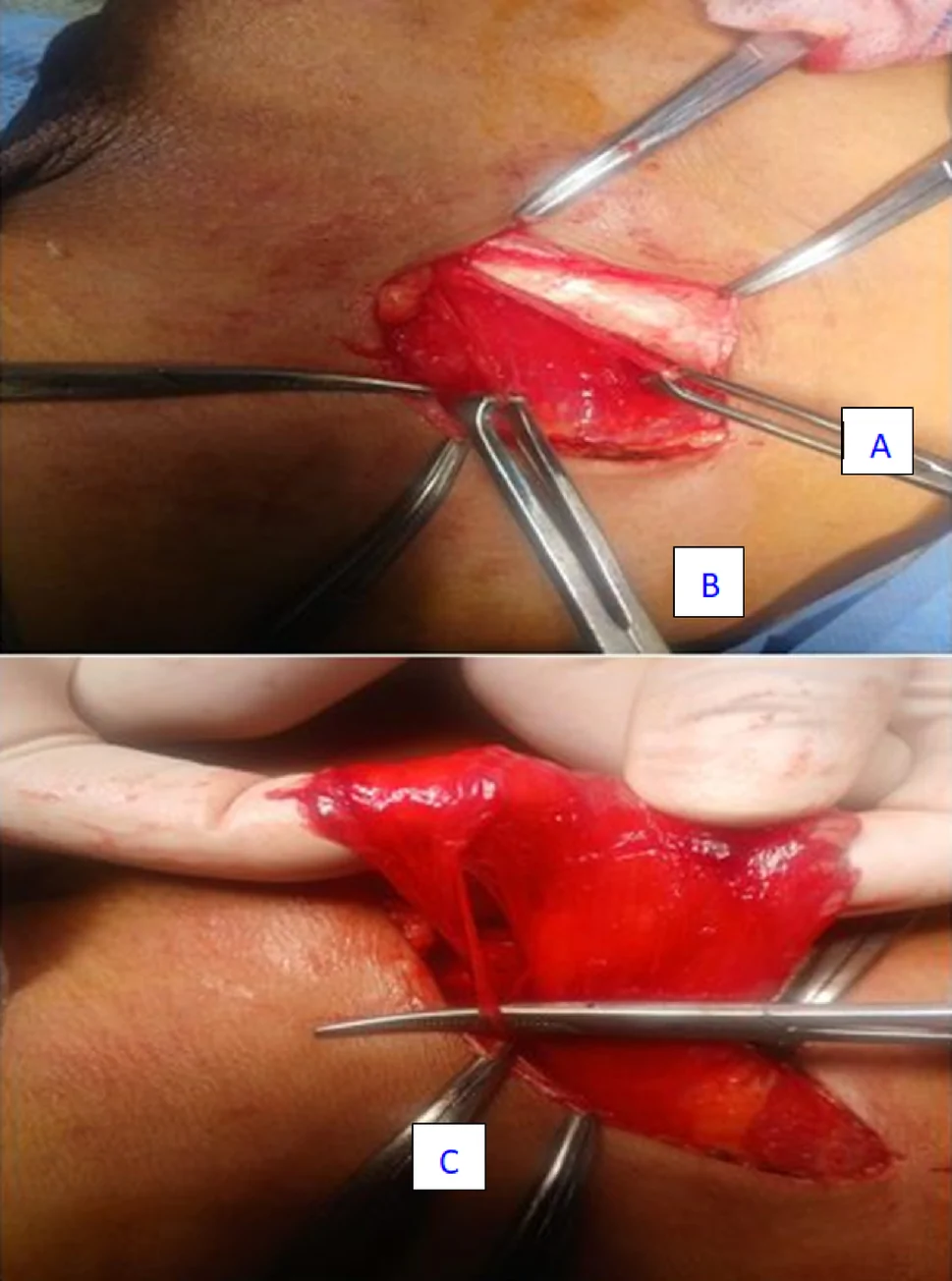
Identification and preservation of **A** iliohypogastric, **B** ilio-inguinal and **C** genital branch of genitofemoral nerves

**Fig. 2:**
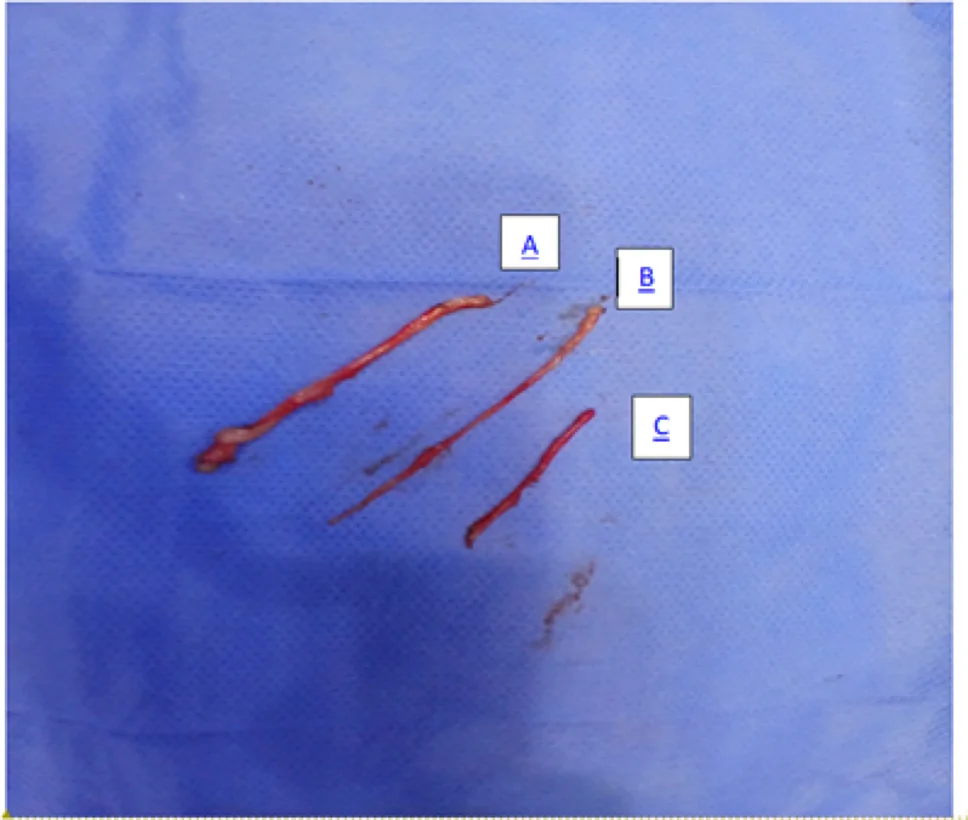
The excised three nerves (**A** iliohypogastric, **B** ilio-inguinal, **C** genital branch of genitofemoral)

In the nerve preservation group, the three nerves were identified and preserved. In the neurectomy group, the ilioinguinal, iliohypogastric, and genital branch of the genitofemoral nerve were identified and dissected, with excision of their entire lengths in the inguinal canal (Fig. 1). Mild traction on the nerves assisted in the trans-macular dissection of their proximal end. This method of nerve dissection led to the disappearance of the proximal end between the muscle fibers, providing no contact between the nerves and the mesh (ilioinguinal and iliohypogastric). The genital branch of the genitofemoral nerve was identified along the course of the spermatic cord and inguinal canal and cut. The nerves were excised using diathermy (Fig. 2).

Postoperatively, all patients administered analgesia in the form of paracetamol at a dosage of 1000 mg every 8 h. Patients received ketorolac injections when needed. Each dose consisted of a 30 mg ampule. The number of ketorolac injections were determined for each patient to evaluate the differences between the two groups concerning the requirement for analgesics in the early postoperative period.

Patients were followed up at the outpatient clinic twice a week for two weeks, then a visit after a month, and another visit after three months to fill in the questionnaire, or the patient might respond to it over a telephone conversation. Patients who were suffering from pain were conservatively managed by NSAIDs. Patients with severe pain and not responding to medical treatment were referred to the pain clinic for local steroid ultrasonic-guided injections.

At the time of responding to the questionnaire, a direct question was given regarding numbness by asking the patient if he had numbness at the groin or the scrotum, with yes/no answer. Patients who reported swelling at the site of surgery were excluded to eliminate recurrence or inflammation as potential causes of pain.

The short-form inguinal pain questionnaire (sf-IPQ) [14] was conducted over a telephone conversation or attendance to assess the presence and severity of postoperative chronic inguinal pain and its effect on patients’ daily activities (Table 1).

**Table 1:** The short-form IPQ (sf-IPQ) questionnaire

1. Estimate the worst pain you felt in the operated groin during this past week: ☐ No pain ☐ Pain present but can be easily ignored ☐ Pain present, cannot be ignored, but does not interfere with daily activities ☐ Pain present, cannot be ignored, interferes with concentration on chores and daily activities ☐ Pain present, cannot be ignored, interferes with best mood ☐ Pain present, cannot be ignored, necessitates bed rest ☐ Pain present, cannot be ignored, prompt medical advice sought
2. If you have experienced groin pain, to what extent has it limited your ability to perform the following activities? *(More than one option may be selected)* ☐ Getting up from a low chair ☐ Sitting down (more than 30 min) ☐ Standing up (more than 30 min) ☐ Going up or down stairs ☐ Driving a car ☐ Exercising or performing sports

The short-form IPQ (sf-IPQ), consisting of two items with a total score ranging between 0 and 12. Question 1 contributes 0–6 points (higher points for more intense pain) and question 2 adds one point for each reported activity limited by pain (6 in all) [14]

The sf-IPQ consists of two items that contribute to a total score ranging from 0 to 12. Question 1 was designed to assess the intensity of pain experienced by the patient. It gives a score ranging from 0 to 6, and the higher the points, the more severe the pain. Question 2 focused on the impact of inguinal pain on the patient’s daily activities. One point was added for each activity limited by pain, with a maximum score of six [14].

Patients with a score of less than three were deemed free, while those with scores of 4–6, 7–9, or 10–12 were classified as having mild, moderate, or severe pain, respectively.

## Results

Between January 2024 and January 2025, 196 male patients were operated on by inguinal hernioplasty. The sample size was consecutively calculated, as the rate of admissions for open inguinal hernia repair was about 20 patients per month. Sixty-nine patients were excluded: either bilateral, recurrent, or huge inguinoscrotal hernia. It was unable to contact seventeen patients. Mean and standard deviation, t test, Chi square test, and Mann–Whitney U test were used to analyze results. Fifty-one patients were operated on with nerve preservation with a mean age of 40.1373 ± 12.70 years, while 59 patients were operated on with triple neurectomy with a mean age of 38.2712 ± 12.42 years, with no statistical difference (*p* value = 0.439) (Table 2).

**Table 2:** Shows age, operative time, sf-IPQ scores, the early postoperative need for analgesics and numbness across both groups

Item						Confidence intervals		*p* value
						Lower bound	Upper bound	
Age				N	Mean ± Std. Deviation			0.439^**^
	Nerve preservation group			51	40.1373 ± 12.70121	− 2.891	6.623	
	Neurectomy group			59	38.2712 ± 12.42194			
Operative time	Nerve preservation group			51	41.33 ± 8.494	− 3.228	3.522	0.931^**^
	Neurectomy group			59	41.19 ± 9.248			
Chronic pain severity with related daily activities	Nerve preservation group	No pain Score (0–3)		40 (78.4%)		0.0E0	0.027	0.008^*^
		Mild pain, score (4–6)		6 (11.8%)				
		Moderate pain, Score (7–9)		4 (7.8%)				
		Severe pain, Score (10–12)		1 (2%)				
	Neurectomy group	No pain, Score (0–3)		56 (94.9%)				
		Mild pain, score (4–6)		3 (5.1%)				
Doses of NSAID	Nerve preservation group		No doses	0		0.0E0	0.027	0.0013^*^
			One dose	45				
			Two doses	3				
			Three doses	3				
	Neurectomy group		No doses	4				
			One dose	55				
			Two doses	0				
			Three doses	0				
Numbness	Nerve preservation group		No	44 (86.3%)				0.641^#^
			Yes	7 (13.7%)				
	Neurectomy group		No	49 (83.05%)				
			Yes	10 (16.95%)				

^**^T test

^*^Mann–Whitney U

^#^Chi square test

The mean operative time in the nerve preservation group was 41.33 ± 8.494 min, while it was 41.19 ± 9.248 min in the neurectomy group, with no significant difference (*p* value = 0.931) (Table 2).

During the first three days after surgery, the need for NSAIDs among patients was lower in the neurectomy group compared to the other group, with a *p* value of 0.0013 (Table 2).

Regarding postoperative chronic pain, eleven patients in the nerve preservation group suffered from pain of varying severity. Six, four, and one patients had mild, moderate, and severe pain, respectively. In the neurectomy group, only three patients had mild pain. There was a significant difference (*p* = 0.008) between the two groups. At the time of the questionnaire, the number of patients suffering from numbness in the nerve preservation group was 7 versus 10 patients in the neurectomy group, which was statistically non-significant (*p* value = 0.641) (Table 2).

## Discussion

Early postoperative pain is typically felt soon after surgery; it can be managed with analgesics and is usually resolved within 15–30 days after surgery without further treatment needed. Conversely, chronic pain, generally persists for three months or more after surgery, may result in a potentially devastating condition and sometimes prevents the patient from performing his normal daily activities [15, 16].

Chronic groin pain is one of the most crippling long-term complications after inguinal hernia repair, which can have a major negative impact on the patient’s satisfaction and quality of life after surgery. This pain may be caused by compression, inflammation, neuroma, or fibrotic reactions induced by surgery and/or the implanted mesh [2, 17, 18] Thus, many surgeons have proposed triple neurectomy (ilioinguinal, iliohypogastric, and genitofemoral) during inguinal hernia repair to minimize the incidence of chronic postoperative pain.

The original contribution of this study was the direct comparison of triple neurectomy to nerve preservation in a randomized, prospective manner to evaluate the short-term incidence of chronic postoperative pain.

In this study, all patients were males; there was no significant age difference between the nerve preservation and neurectomy groups. McIntosh and his colleagues [19] found a male predominance in the inguinal hernia incidence of 9:1. This higher incidence may be attributed to the larger inguinal canal in males. Also, the testicles descent from the abdominal cavity to the scrotum before birth increases the liability for inguinal hernia occurrence. Moreover, inguinal hernia incidence, especially the direct one, increases with aging due to muscle weakness and increased chances of prostatic enlargement [20].

In the present study, there was no difference between both groups regarding the operative time. This means that neurectomy is not a time-consuming procedure.

Patients in the triple neurectomy group had a reduced requirement for ketorolac injections compared to the nerve preservation group. This presupposed that neurectomy reduced early postoperative pain and diminished the requirement for NSAIDs. All patients were on regular paracetamol therapy and ketorolac injection was on demand.

In our work, chronic postoperative inguinal pain was higher in the nerve preservation group at the time of the questionnaire, which was evaluated at least three months postoperatively. 21.5% in the nerve preservation group had different degrees of pain compared to 5.1% in the neurectomy group. Also, the intensity of pain was higher in the nerve preservation group; six, four, and one patients experienced mild, moderate, and severe pain respectively, according to the sf-IPQ in the nerve preservation group. In the neurectomy group 94.9% experienced no pain and only 5.1% had mild pain.

Numbness was evaluated by a direct question. 13.7% of patients in the nerve presentation group experienced numbness versus 16.9% in the neurectomy group. Although it seems that numbness was more in neurectomy group, this difference was not statistically significant and did not affect the daily activities.

Patients with mild pain tolerated it without further treatment being required. Patients with moderate pain sometimes require analgesics for pain control. The patient had severe pain; the pain affected his daily activities even with analgesics. The patient sought medical advice and was referred to the pain clinic. He received local steroid ultrasonic-guided injections for three doses, once per week, with mild relief and the patient was prepared for ultrasonic-guided pulsed radiofrequency ablation of the ilioinguinal and iliohypogastric nerves at the time of writing this paper. Similarly, Dittrick et al. [21] found that routine ilioinguinal neurectomy was associated with a significantly lower incidence of postoperative chronic pain when compared to nerve preservation in a retrospective review of ninety patients who underwent Lichtenstein repair of inguinal hernia with either preservation or elective division of the ilioinguinal nerve. Jíšová et al. [22] concluded that triple neurectomy is a successful treatment for inguinodynia.

In a study conducted by Ravichandran et al. [23] assessing ilioinguinal nerve division in patients with bilateral inguinal hernia, one side was operated on by nerve preservation, and the other side by ilioinguinal neurectomy, where the patient acted as their own control. His study found no evidence to support ilioinguinal nerve division. However, the small sample size was a limiting factor for this study, and so generalization of these results cannot be done.

The ilioinguinal nerve gives sensory innervation to the skin over the groin area and upper thigh. Nerve excision may induce numbness in this area [23]. This study showed no statistically significant difference between the two groups regarding numbness at the time of the questionnaire, which was at least 3 months postoperatively. This may be due to compensation by cross-innervations from nearby cutaneous nerves. Similarly, Mui et al. [24] demonstrated that excision of the ilioinguinal nerve was not associated with additional morbidities regarding neurosensory disturbances, numbness, or quality of life at the 6-month follow-up.

Samarajeewa and his colleagues [25] reported in their case report study that a 54-year-old man with intractable left-sided groin pain for 3 years after recurrent inguinal hernia repair failed to relieve pain with analgesics and nerve block, but the results were satisfying after laparoscopic triple neurectomy.

In a meta- analysis study performed by Barazanchi et al. ilioinguinal and iliohypogastric neurectomy during Lichtenstein hernioplasty was found to reduce the chronic postoperative pain [26].

## Conclusion

Prophylactic triple neurectomy provides the advantage of decreasing chronic postoperative pain after open inguinal hernia surgery. While our findings are promising, further studies with larger populations are needed before recommending triple neurectomy as a standard step during inguinal hernioplasty.

### Limitations

The relatively small sample size restricts the strength of our conclusions regarding the recommendation of routine use of prophylactic triple neurectomy during open inguinal hernia repair.

### Recommendations

Further prospective studies with larger cohorts are warranted to validate the efficacy of triple neurectomy in reducing chronic postoperative pain and to support its potential standardization in surgical practice.

## Data Availability

The datasets generated during the current study are available from the corresponding author on reasonable request.
